# Effect of earthworms on mycorrhization, root morphology and biomass of silver fir seedlings inoculated with black summer truffle (*Tuber aestivum* Vittad.)

**DOI:** 10.1038/s41598-021-85497-8

**Published:** 2021-03-17

**Authors:** Tina Unuk Nahberger, Gian Maria Niccolò Benucci, Hojka Kraigher, Tine Grebenc

**Affiliations:** 1 Slovenian Forestry Institute, Večna pot 2, 1000 Ljubljana, Slovenia; 2 Department of Plant, Soil, and Microbial Sciences, Michigan State University, 426 Auditorium Road, East Lansing, MI 48824 USA

**Keywords:** Ecology, Plant sciences

## Abstract

Species of the genus *Tuber* have gained a lot of attention in recent decades due to their aromatic hypogenous fruitbodies, which can bring high prices on the market. The tendency in truffle production is to infect oak, hazel, beech, etc. in greenhouse conditions. We aimed to show whether silver fir (*Abies alba* Mill.) can be an appropriate host partner for commercial mycorrhization with truffles, and how earthworms in the inoculation substrate would affect the mycorrhization dynamics. Silver fir seedlings inoculated with *Tuber. aestivum* were analyzed for root system parameters and mycorrhization, how earthworms affect the bare root system, and if mycorrhization parameters change when earthworms are added to the inoculation substrate. Seedlings were analyzed 6 and 12 months after spore inoculation. Mycorrhization with or without earthworms revealed contrasting effects on fine root biomass and morphology of silver fir seedlings. Only a few of the assessed fine root parameters showed statistically significant response, namely higher fine root biomass and fine root tip density in inoculated seedlings without earthworms 6 months after inoculation, lower fine root tip density when earthworms were added, the specific root tip density increased in inoculated seedlings without earthworms 12 months after inoculation, and general negative effect of earthworm on branching density. Silver fir was confirmed as a suitable host partner for commercial mycorrhization with truffles, with 6% and 35% mycorrhization 6 months after inoculation and between 36% and 55% mycorrhization 12 months after inoculation. The effect of earthworms on mycorrhization of silver fir with *Tuber aestivum* was positive only after 6 months of mycorrhization, while this effect disappeared and turned insignificantly negative after 12 months due to the secondary effect of grazing on ectomycorrhizal root tips.

## Introduction

Hypogeous fungi are a diverse polyphyletic group of over 150 globally distributed genera ^1^ that form sequestrate fruiting bodies ^2,3^. Among hypogeous fungi, truffles, the genus *Tuber* P.Micheli ex F.H.Wigg., family Tuberaceae ^4^, are recognized as a gastronomic delicacy due to their unique species-specific aromas ^5–7^. Due to this characteristic, truffles are a significant commercial product among non-timber forest products ^8^.

Truffles are ectomycorrhizal ^9^, ectendomycorrhizal ^10^ or endophytic fungi ^11^ that are unable to complete their life cycle without a symbiotic relationship with a vital plant host ^3,12^. Truffles grow in an ectomycorrhizal symbiosis with most boreal and temperate trees, and with perennial shrubs of the northern hemisphere, such as oaks, hazel, beech, birch, pines, pecan, cistus, sunrose, etc. ^13–17^. The only known example of forming ectendomycorrhizae is with the strawberry tree ^10^. High cultural, gastronomic, and economic interest in truffles guided many attempts of truffle cultivation. However, cultivation of commercially valuable species has gained higher interest only in the last few decades ^13,14,18^. Today we can successfully cultivate most commercially valuable truffle species with a range of host plants using truffle-inoculated seedlings ^19,20^. A routine spore-inoculation process of seedlings in greenhouses has remained mainly unchanged for decades ^21^. A pasteurized substrate with added pre-treated truffle spores is used for inoculation of seedlings. Seedlings are then maintained in nurseries until ectomycorrhizae are established ^21–23^. Current truffle mycorrhization procedures do not suggest additional treatments such as microbiota or pedofauna enrichments of substrate, nor was this ever tested under nursery conditions (*ibid.*).

The truffle life cycle is limited to soil ^24^. While mycelia actively grow in soil, mycorrhizas move mainly together with the roots, and soil surface conidia are wind dispersed ^25^, sexual spores move by soil fauna, such as large and small mammals, insects, earthworms, etc. for dispersal ^26^. Gange ^27^ found that earthworms graze preferentially on soils containing mycorrhizal fungal propagules and fruiting bodies, and disperse propagules and spores by soil ingestion or as particles attached to their cuticles ^28^. Earthworms profoundly impact the symbiosis between mycorrhizal fungi and plants not only directly by grazing and moving fungal propagules in soil but also indirectly via changing soil permeability and modifying nutrient availability ^28–31^. In nature earthworms preferentially feed on fungal mycelia, consequently affecting the mycelium either by disrupting the contact of the external hyphae from the roots, decreasing fungal biomass in soil or reducing hyphal length in substrate ^28,29^.

*Tuber aestivum* Vittad. (the summer truffle) is a truffle species with a very broad ecological niche ^32^ and consequently one of the most geographically widespread truffles in Europe ^2,18,33^. *Tuber aestivum* is also one of the less demanding species that forms ectomycorrhizae with a broad range of ectomycorrhizal partners ^13,34^ thus making it easy and successfully cultivated widely, in Europe as far north as Sweden and Finland ^14,21^. Although *Tuber magnatum* Pico (the white Italian truffle) is regarded as the most famous and expensive truffle, in Italy alone over 65% of the traded and processed truffles are *Tuber aestivum* ^35^.

Silver fir (*Abies alba* Mill.) is an ectomycorrhizal evergreen conifer of mountainous regions of eastern, western, southern, and central Europe ^36^. Silver fir is a long-lived tree with of great ecological and silvicultural value (*ibid*.) known to form ectomycorrhizae with a range of fungal genera. Past studies revealed the presence of the genus *Tuber* in mycorrhizae with silver fir, but infrequently and in low percentages ^37–40^. Silver fir in this study was selected as a plant partner tree due its ability to form ectomycorrhizae with truffles in nature, which has never been confirmed in a nursery inoculation process, and never with a commercial truffle species.

Based on the roles of earthworms in soil, and contradictory statements about their effects on mycorrhization, we performed a greenhouse inoculation experiment to analyze their effects on mycorrhization and fine root growth. For this purpose, we inoculated silver fir seedlings with *Tuber aestivum* to test if silver fir could be an adequate ectomycorrhizal host for commercial truffles. We set the following hypotheses: (1) spore inoculation, earthworms or a combination of both will have a significant effect on silver fir root biomass and morphology; (2) silver fir can be an adequate symbiotic partner for commercial production of *Tuber aestivum* mycorrhized seedlings; and (3) earthworms added to the mycorrhization system will have a positive effect on mycorrhization levels of silver fir with the commercial truffle species *Tuber aestivum* under greenhouse conditions.

## Materials and methods

### Pot experimental design

Silver fir seedlings were obtained from the LIECO nursery (LIECO GmbH & Co KG, Kalwang, Austria) and were approximately four months old at the beginning of the experiment. Seedlings of about the same size and with well-developed above and belowground parts were used for the experiment. A check of randomly selected ten silver fir seedlings confirmed the absence of any contaminant ectomycorrhizal fungi prior to inoculation.

The substrate for mycorrhization used in the experiment was adapted from ^21,41^. The final volumes of pasteurized components in substrate mixture was: black peat (50 vol.%) (Agrocentre Roko d.o.o., Hoče, Slovenia), vermiculite (33 vol.%) (Njiva d.o.o., Ložnica pri Savinje, Slovenia), perlite (17 vol.%) (Knauf Gips KG, Iphofen, Germany). and ground limestone (2 vol.%) (Rotar d.o.o., Dobrova, Slovenia). Prior to adding *Tuber aestivum* spore suspension, substrate was well watered, and pH was adjusted with additional lime dust to a range from 7.0 to 7.5.

Inoculation substrate was supplemented with a truffle spore suspension containing 2 g fresh weight of mature truffle gleba per seedling^2^. Fresh truffles were washed, surface sterilized in 70% ethanol for 10 min and washed under running tap water for 20 min. Finally, peridium was peeled and gleba was kept frozen (− 20 °C) until preparation of the suspension. Only high quality, fully ripened (> 95% asci with ripened spores), mechanically undamaged, and unrotten sporocarps of *Tuber aestivum* were used. Quality and identification of each sporocarp was confirmed by a truffle specialist, and reference sporocarps are kept in the LJF herbarium under accession numbers TUBAES/290915 (under hornbeam and oak; Žlebič, Slovenia), TUBAES/231114A (under beech and silver fir; Snežna jama, Slovenia) and TUBAES/050714A (under beech and silver fir; Mavrovo, North Macedonia). To obtain the desired concentration of 2 g *Tuber aestivum* spores per plant, 200 g of sporocarps were homogenized with a surface sterilized blender in sterilized water. The spore suspension was added to substrate in adequate volume and the inoculation substrate was mixed thoroughly by hand. 25 silver fir seedlings were planted in individual containers with dimensions 52 cm × 38 cm × 50 cm. These containers were used instead of traditional pots (340 ml/650 ml) pots to ensure sufficient living space for added earthworms since traditional pots were proved insufficient for their survival. For the container experiment we established four treatments: control treatment, treatment with added truffle inoculum, treatment with added earthworms, and treatment with a combination of truffle inoculum and earthworms. One earthworm per seedling was added approximately one month after the experiment was initiated. *Eisenia fetida* (Savigny, 1826) earthworms were obtained from a local farm in Slovenia, where animals of approximately the same size (ca 4 cm long) were selected for the experiment. Containers with different treatments were kept in a fully controlled greenhouse for one year. Controlled conditions with a day light regime and temperature 22 °C for 16 h and a night regime and temperature of 15 °C for 8 h were maintained throughout the experiment.

### Evaluation of mycorrhization levels and assessment of fine root morphology

Fine root mycorrhization was evaluated 6 and 12 months after inoculation, which is the standard time for mycorrhization success control ^19^. Ten seedlings were randomly selected from each treatment per evaluation time and carefully removed from containers. Whole root systems of selected plants were washed in tap water, fine roots were separated and placed in trays filled with distilled water and scanned by an Epson Perfection V700 Photo scanner (Seiko Epson Corp., Suwa, Nagano, Japan). Fine roots were defined as roots with diameter < 2 mm ^42^. Scans were further analyzed using WinRHIZO software (Regent Instruments Inc., Québec City, Canada) following the protocol in ^43^. The total root length, number of root tips, and number of branches per individual seedling were assessed and the following rations were calculated: (1) fine root length: fine root biomass (SRL—specific root length), (2) number of root tips: root biomass (root tip density), (3) number of root tips: fine root length (specific root tip density), and (4) number of branches: fine root biomass (branching density). Fine root systems biomass was assessed (SCALTEC SBC-31 balance; Denver Instrument, Bohemia, NY, USA) after drying at 70 °C.

Ectomycorrhizae from each of 100 seedlings were identified following morphological criteria in ^44–46^ under a binocular (Olympus SZH, Tokyo, Japan) and a microscope (Zeiss AxioImager Z2, Carl Zeiss Microscopy GmbH, Jena, Germany) following the procedure described in Fischer and Colinas ^47^. First the complete root system was cut longitudinally, and one half was chosen for examination. Roots from the chosen half were further cut in 2–3 cm segments and placed over a 1 cm × 1 cm grid in a large petri dish filled with distilled water. In consecutively random chosen grid squares, all fine roots were separated into non-mycorrhizal root tips (N), *Tuber aestivum* ectomycorrhizal root tips (T) and contaminant ectomycorrhizal root tips (O). Ectomycorrhizal tips in each category were counted until the total sum per seedling reached 250 ectomycorrhizal tips. Old and senescent ectomycorrhizal tips were categorized as non-mycorrhizal root tips. Based on 250 counted ectomycorrhizal tips the proportion of *Tuber aestivum* ectomycorrhizae (PT = T/(total number of ectomycorrhizal tips)), and the proportion of contaminant ectomycorrhizae (PO = O/(total number of ectomycorrhizal tips)) were calculated.

Seedlings from each treatment and for each sampling time were subjected to a standardized quality control certification procedure to evaluate the suitability of the batch for commercialization. The quality control followed ^48^, according to which a certified plant must show at least 25% of all fine roots mycorrhized with the desired truffle species, and the level of contaminant ectomycorrhizal fungi must not exceed 25% of all fine roots. Seedlings from each treatment and for each sampling time were also evaluated following criteria proposed by Fischer and Colinas ^47^, where the minimum colonization by the desired truffle species should be at least 10% of all fine roots, with a level of contaminant ectomycorrhizal fungi not higher than 50%.

### Molecular analysis

Most truffle mycorrhized certification procedures also require a DNA-based confirmation of the ectomycorrhiza identity ^19^. To fulfill this criterion, ectomycorrhizal tips from different morphotypes were subjected to molecular identification. DNA extractions were performed with a DNeasy Plant Mini kit (Qiagen, Hilden, Germany) following manufacturer’s instructions. The nuclear rDNA ITS region was amplified from isolated DNA using the fungus specific primer pair ITS1F and ITS4 ^49,50^ and ITS5 and ITS7 ^51^. The PCR reactions with the primer pair ITS1f/ITS4 were performed as described in Grebenc and Kraigher ^52^, while the PCR reactions with the primer pair ITS5/ITS7 were carried out under the following conditions: a denaturation step of 7 min at 95 °C, followed by 32 cycles of 15 s at 94 °C, 15 s at 56 °C and 40 s at 72 °C, with a final step at 72 °C for 7 min. PCR products were run on 1.5% agarose gels in 0.5 × TBE buffer and visualized with Gel Doc EQ System, PC (BioRad, ZDA). Amplified DNA fragments were cut out of agarose gels and purified with the innuPREP DOUBLEpure Kit (Analytik Jena AG, Jena, Germany) following manufacturer’s instructions. After the DNA fragments purification, sequencing was performed at the commercial sequencing laboratory Macrogen (Macrogen Europe B.V., Amsterdam, The Netherlands). All samples were sequenced in both directions with the primers ITS1f/ITS4 ^49,50^ or ITS5/ITS7 ^51^. The obtained sequences were processed in Geneious version 11.1.4 (https://www.geneious.com, Kearse et al. ^53^. The BLASTN algorithm from the NCBI website (National Center for Biotechnology Information; https://blast.ncbi.nlm.nih.gov/Blast.cgi) was used to assess the similarity of obtained ITS sequences to sequences in GenBank. Representative sequences from ectomycorrhizal root tips were submitted to GenBank.

### Statistical analysis

All statistical analyses were performed with R 3.5.1 ^54^. Normal distribution and homogeneity of variance were tested with the Shapiro–Wilk normality test and the Bartlett test, and were improved with the approach of Tukey’s Ladder of Powers where needed (p > 0.05), from the package “rcompanion”. For statistical evaluation of differences between treatments, two-way analysis of variance (ANOVA) was applied to test the effects of time from spore inoculation and treatment description as independent factors and their interaction. Further one-way ANOVA was used to analyze the effects of added inoculum, earthworms, and the combination of both on root biomass and root morphology, separately per time from truffle spore inoculation. As a post-hoc test, the Tukey HSD test was used. The effect of earthworms on mycorrhization with *Tuber aestivum* was tested with Student’s t-test using a two-sample unequal variance (heteroscedastic), after the normal distribution was confirmed and data variance of both treatments was confirmed as significantly different after an F test.

## Results

### Effects of mycorrhization, earthworms or combination of both treatments on silver fir fine root biomass and morphology

The different inoculation treatments revealed contrasting effects on fine root biomass and morphology of silver fir seedlings. Only a few of the assessed parameters showed statistically significant different effects when compared to the performance of the control plants (Supplement Table 1).

**Figure 1:**
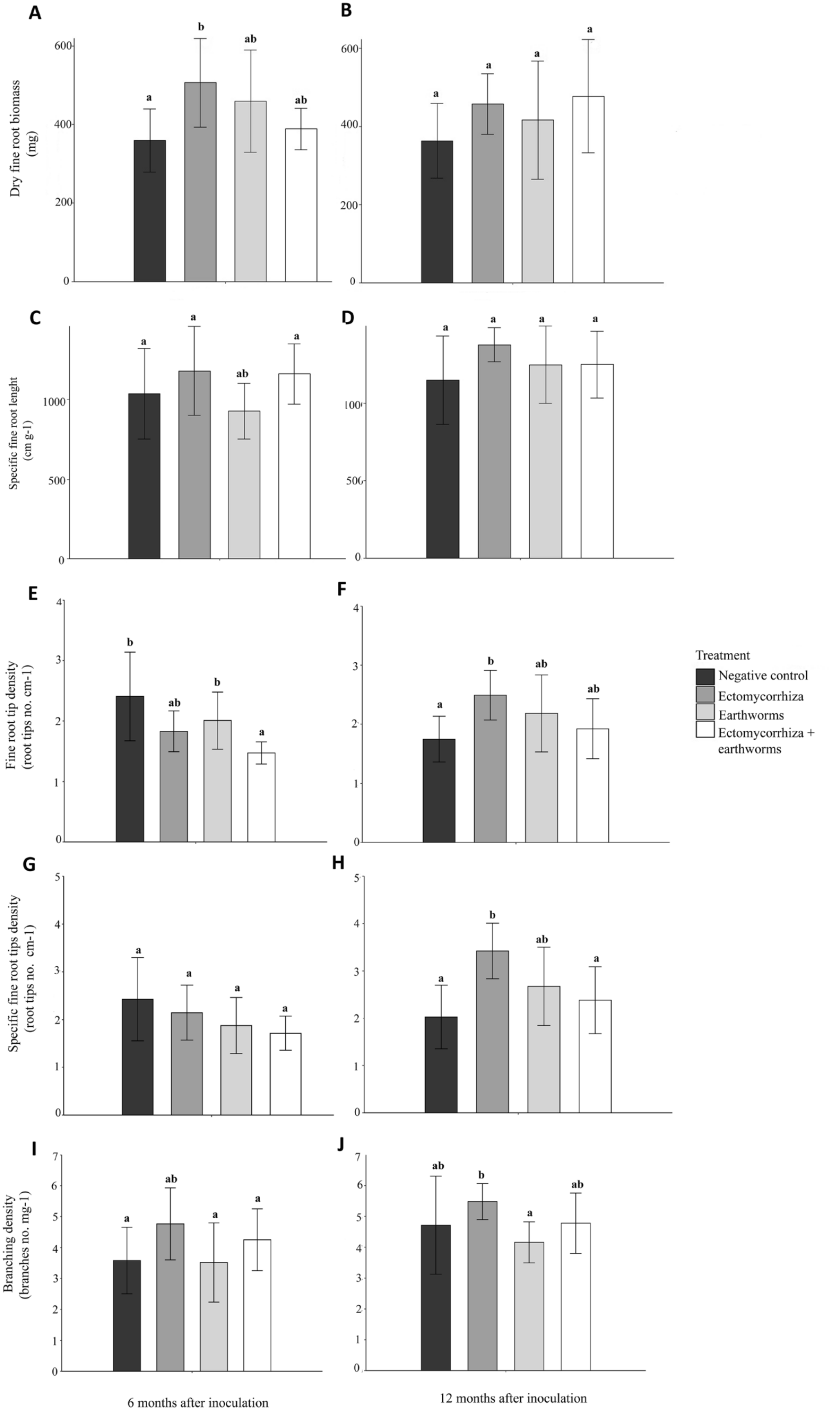
Effects of mycorrhization with *Tuber aestivum*, *Eisenia fetida* earthworms and a combination of both treatments on silver fir root biomass and fine root morphology parameters after 6 and 12 months of mycorrhization in containers under controlled conditions; n = 10. The significantly different values after the Tukey HSD post-hoc test (p < 0.05) are annotated with a different lowercase letter.

Mycorrhization with *Tuber aestivum*, addition of *Eisenia fetida* earthworms to mycorrhization substrate, and the combination of both treatments resulted in significant differences in fine root morphology and biomass (Fig. 1). Fine root biomass was significantly higher only in *Tuber aestivum* mycorrhized plants 6 months after inoculation (Fig. 1A). This effect became insignificant after 12 months, and only in seedlings mycorrhized with *Tuber aestivum* and with the addition of *Eisenia fetida* a pronounced increase was noted (Fig. 1B). The Tukey HSD test showed no significant differences in specific fine root length neither 6 nor 12 months after inoculation (Fig. 1C,D, respectively). The fine root tip density was significantly lower in earthworm- and truffle-inoculated seedlings 6 months after inoculation (Fig. 1E). After 12 months in control, the fine root tip density decreased, and highest number of fine root tips was observed in *Tuber aestivum* mycorrhized plants (Fig. 1F). Specific root tip density 6 months after inoculation was highest in control plants and insignificantly dropped in all other treatments (Fig. 1G). 12 months after inoculation the specific root tip density remained the same in control plants while it increased significantly in inoculated seedlings and insignificantly in treatments involving earthworms (Fig. 1H). The ratio between number of branches and fine root biomass expressed as branching density (number of root forks/mg of fine roots) was higher in both treatments where silver fir seedlings were inoculated with *Tuber aestivum*. 6 months (Fig. 1I) and 12 months (Fig. 1J) after inoculation, the highest number was calculated for treatments with *Tuber aestivum* mycorrhized seedlings followed by combined treatment of *Tuber aestivum* and earthworms. A negative effect of earthworm grazing on branching density was also observed between *Tuber aestivum* mycorrhized seedlings and seedlings that were inoculated solely with earthworms.

### Silver fir as a host for the commercial truffle species *Tuber aestivum*

**Figure 2:**
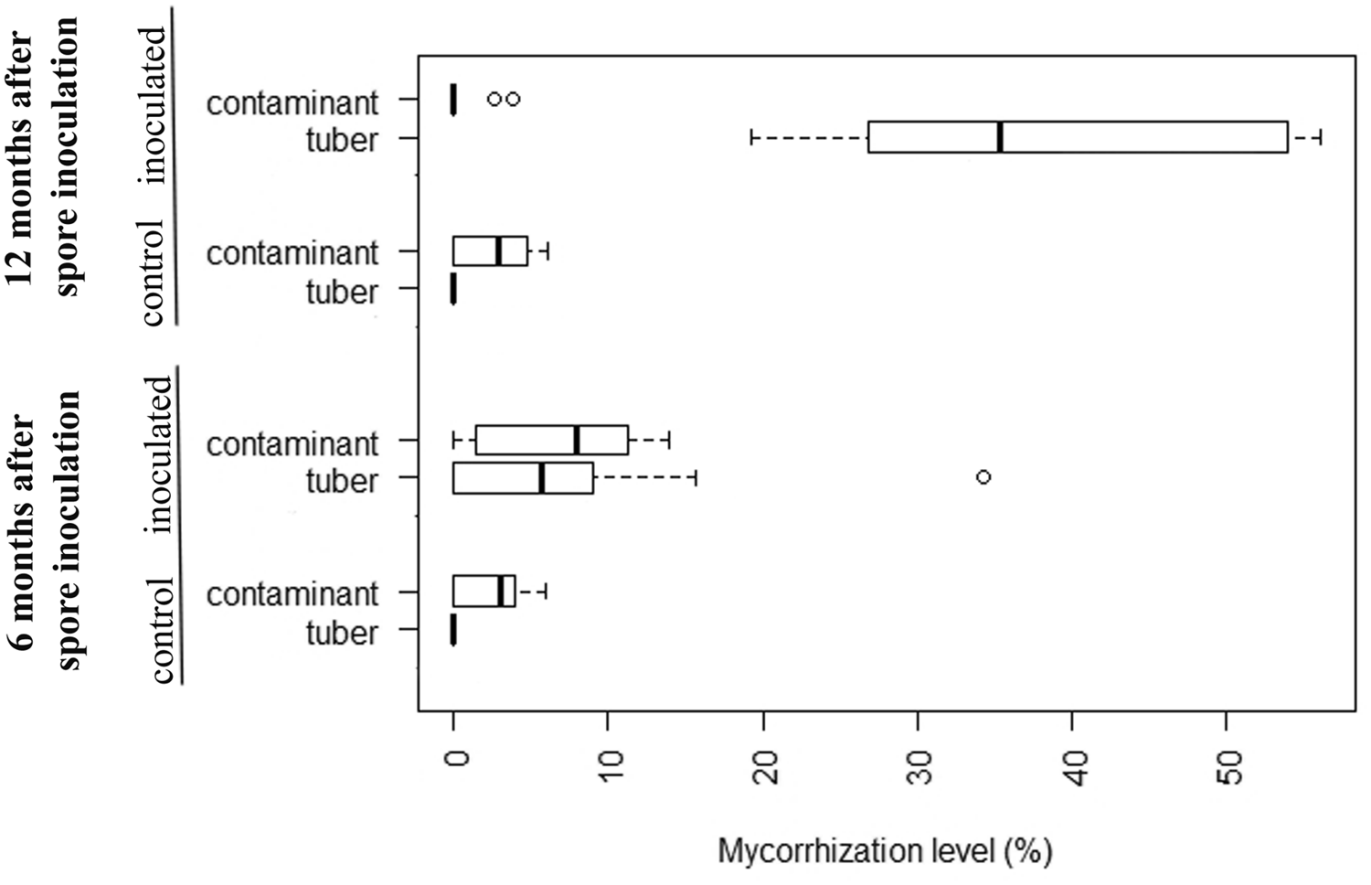
Mycorrhization levels (%) of silver fir plants inoculated with *Tuber aestivum* and control plants, 6 and 12 months after the start of the experiment. The figure represents a boxplot based on % of mycorrhized fine roots based on ten seedlings per treatment. Outliers are shown with circles.

Mycorrhization levels and quality control of silver fir plants mycorrhized with *Tuber aestivum* were checked 6 and 12 months after spore inoculation. The mycorrhization level distribution falls between 6% and 35% (mean = 6.47% ± 5.1%) 6 months from inoculation and between 36% and 55% (mean = 37.72% ± 14.04%) 12 months from inoculation. The level of contaminant ectomycorrhizae 6 months after spore inoculation was low, between 7% and 13% (mean = 8.01% ± 5.05%) in *Tuber aestivum* inoculated seedlings. 12 months after spore inoculation the level of contaminant fungi in *Tuber aestivum* inoculated seedlings was even lower, between 0% and 3% (mean = 0.65% ± 1.2%) for 50% of all truffle inoculated seedling samples (Fig. 2).

**Figure 3:**
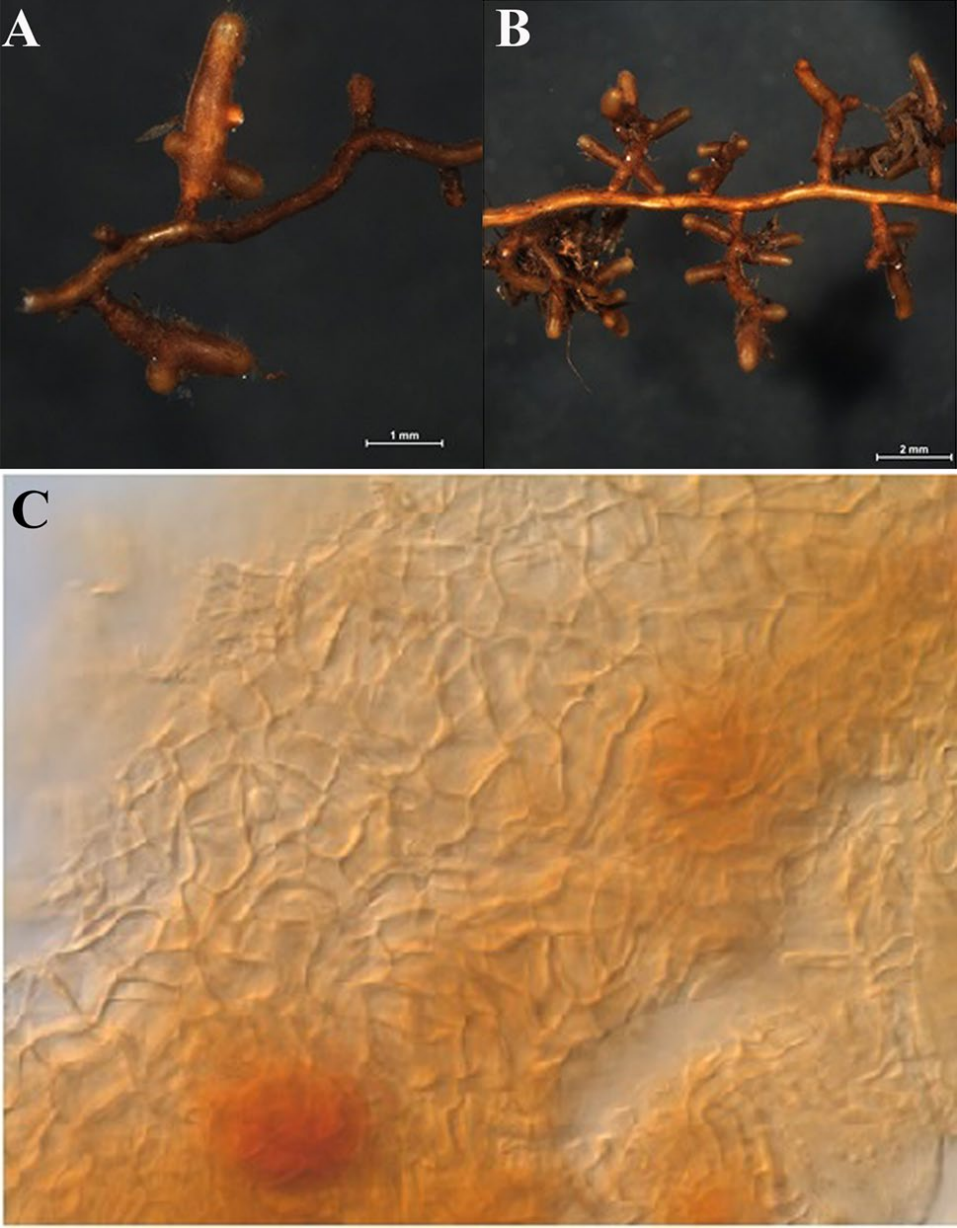
*Tuber aestivum* root tips under dissecting microscope (**A**, **B**) and root tip outer-middle mantle under microscope (**C**).

Mycorrhization levels of seedlings inoculated with *Tuber aestivum* evaluated by ^48^ criteria for a truffle inoculated seedlings certification revealed that 6 months after inoculation 20% of *Tuber aestivum* inoculated silver fir seedlings passed the criteria of a minimum 25% of mycorrhized root tips. 12 months after spore inoculation, the share of *Tuber aestivum* inoculated silver fir seedlings passing the minimum required criterion was 80%. Considering the criteria proposed by Fischer and Colinas^47^ all silver fir seedlings inoculated with *Tuber aestivum* met criteria 12 months after spore inoculation (Fig. 2). All inoculated seedlings showed well-developed *Tuber aestivum* ectomycorrhizae (Fig. 3A,B) with typical pseudoparenchymatous outer mantle layers in the ectomycorrhizal mantle (Fig. 3C). Sequences from morphotypes showed 100% identity with *Tuber aestivum*/*uncinatum*, and contaminating ECM belonging to the genus *Telephora* are available in GenBank under accession numbers MN270339–MN270351 and MN270390–MN270412.

### Effect of earthworms on mycorrhization with *Tuber aestivum*

**Figure 4:**
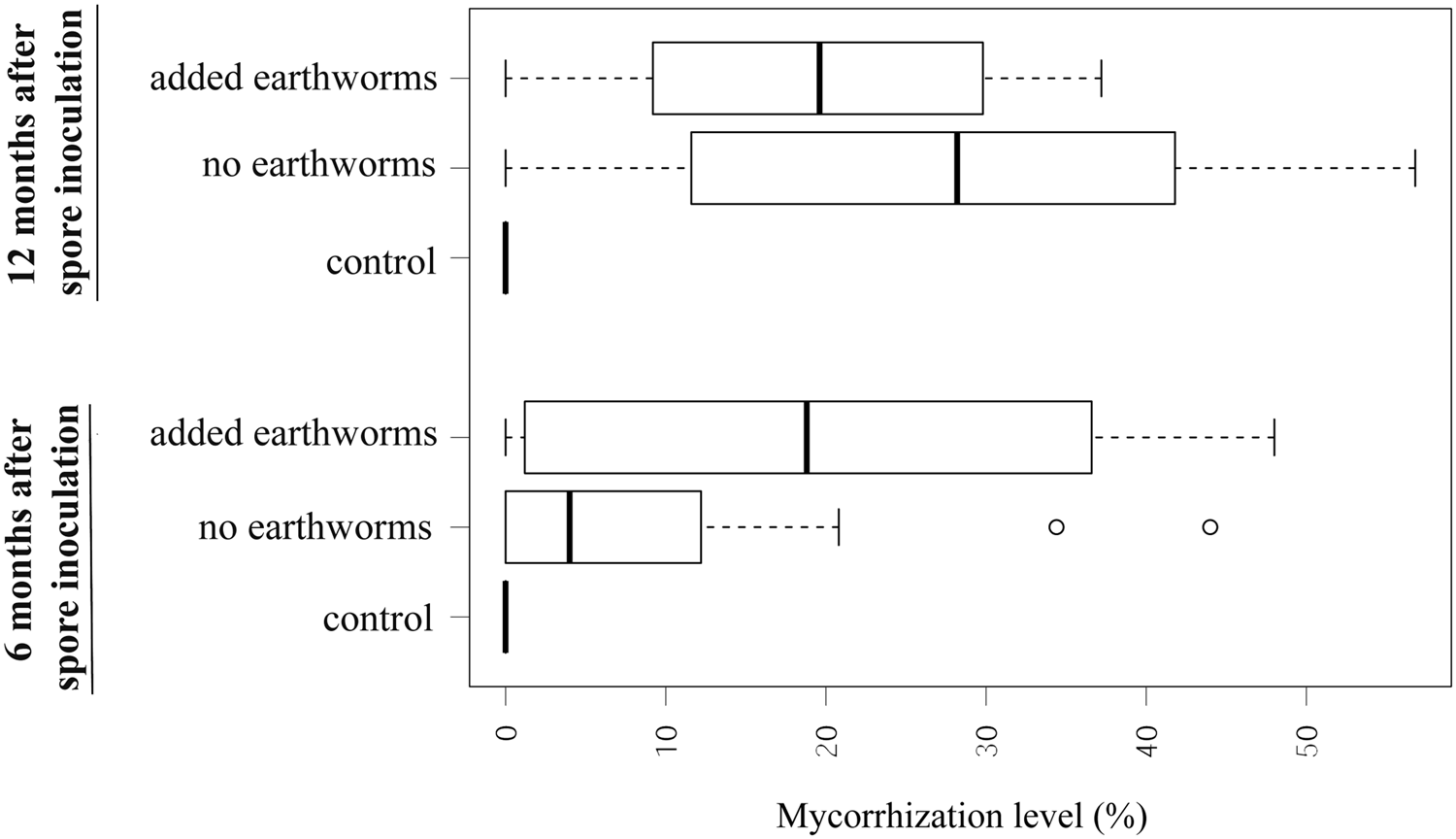
Mycorrhization level of *Tuber aestivum* inoculated seedling with and without added earthworms, after 6 months and 12 months from spore inoculation, n = 20.

**Figure 5:**
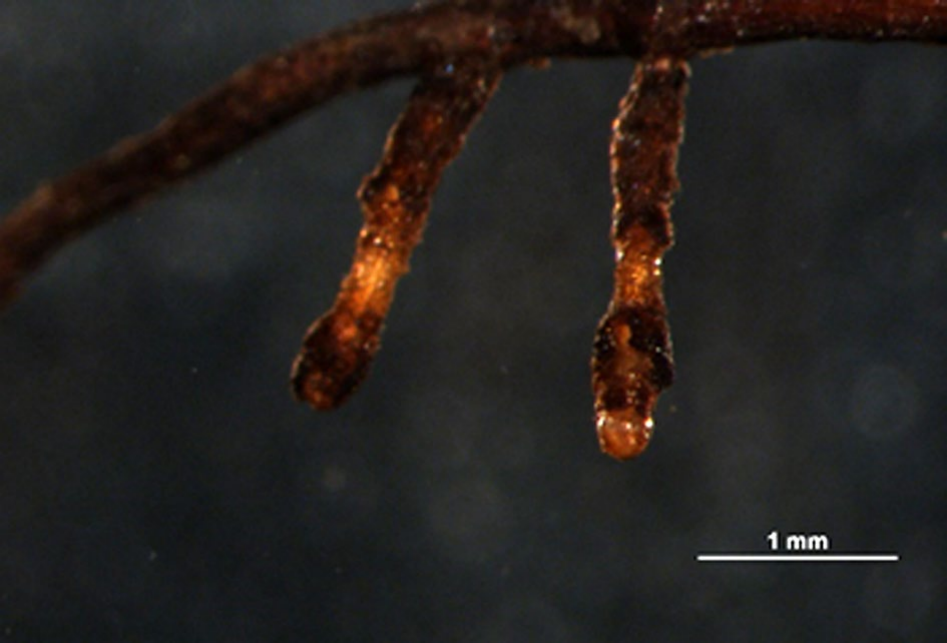
Consequences of *Tuber aestivum* mycorrhized ectomycorrhizal fine roots grazed by earthworms. Ectomycorrhizal fine roots which were partially or completely grazed were regarded as old and senescent ectomycorrhizal root tips and were counted as non-mycorrhizal root tips.

The addition of earthworms to *Tuber aestivum* inoculated silver fir seedlings (Fig. 4) resulted in a significantly higher mycorrhization percentage in seedlings 6 months after inoculation (Student’s t-test p = 0.025; F test for homogeneous variances p = 0.125, kurtosis = 1.809 (no earthworms)/− 1.458 (with earthworms) and skewness = 1.542 (no earthworms)/0.268 (with earthworms)). 12 months after inoculation the effect of added earthworms turned into insignificantly negative (Student’s t-test p = 0.096; F test for homogeneous variances p = 0.117, kurtosis = − 1.115 (no earthworms)/− 1.319 (with earthworms) and skewness = 0.072 (no earthworms)/− 0.108 (with earthworms)). The reduction of the mycorrhization level in 12-month-old seedlings was at least partially due to grazing of ectomycorrhizal fine roots by earthworms (Fig. 5) that was not observed earlier in the experiment. All negative control plants showed zero mycorrhization level with *Tuber aestivum*.

## Discussion

### *Tuber aestivum* spore inoculation effect on fine root biomass and morphology

Effects of ectomycorrhizal fungi on fine roots morphology and branching ^55–60^ are well established, but very few studies have included effects of earthworms on fine roots and mycorrhization. Our results recoded 6 and 12 months after silver fir inoculation with ectomycorrhizal fungi and earthworms did not give straightforward results. The fine root biomass increase was significant only for mycorrhized seedlings without earthworms in first 6 months. Similar phenomenon was already observed by while the insignificant positive effect of earthworms could be explained by their active roles in nutrient mixing and nutrient-releasing effects ^61^. As another early effect, the fine root tip density was higher in control plants 6 months after inoculation and significantly lower in earthworm-inoculated plants. This early peak is explained by the relatively little time that passed from inoculation to analysis by ectomycorrhizal fungi and/or earthworms. These results indicate that the *Tuber aestivum* mycorrhization process in silver fir is among slower ones, similar to mycorrhization of non-typical *Tuber aestivum* ectomycorrhizal host plants, such as pecan ^6,23^, and in contrast to more pioneer species like hazel that are mycorrhized faster ^62,63^. The slower mycorrhization of silver fir with *Tuber aestivum* was also confirmed by the failure to fulfill the certification criteria 6 months after inoculation of seedlings. On the other hand, 12 months after inoculation, the lowest number for fine root tip density was expectedly observed in control plants where no branched ectomycorrhizal roots were formed, in contrast to *Tuber aestivum* mycorrhized plants with abundant monopodial-pinnate to monopodial-pyramidal branching (Fig. 3A,B). Another parameter with a significant change was the specific fine root tip density 12 months after inoculation. Specific fine root tip density was higher in mycorrhized seedlings only, and not in treatments with added earthworms, which is again related to the changed morphology of ectomycorrhizal fine roots compared to a non-mycorrhized fine roots ^64^.

All other morphological parameters, regardless of the duration of experiment, showed no significant deviation from control plants and among parameters. The low responsiveness of morphological root parameters to mycorrhization with *Tuber aestivum* and earthworms is due to silver fir being a slow-growing, shade-tolerant long-living conifer tree species of predominantly mountainous regions ^36^, opposite to *Tuber aestivum* as a fast-growing pioneer species of ruderal areas with dynamic soil conditions^18,32,65^. We have also recorded an insignificant effect of earthworms on fine root biomass of non-mycorrhized seedlings. This “lack of interest” of earthworms in bare fine roots is likely due to the presence of repelling resins in fine roots as in other parts of silver fir ^66,67^.

### Silver fir is an appropriate host partner for *T. aestivum* cultivation

We have successfully established ectomycorrhizae of *Tuber aestivum* on silver fir in greenhouse conditions, using a standard commercial inoculation procedure ^21,23,41^. Ectomycorrhizae of *Tuber aestivum* with silver fir had not been confirmed before and we managed to produce seedlings that passed two common procedures for certification of truffle-inoculated seedlings ^47,48^ at least after 12 months of mycorrhization. The successful mycorrhization with *Tuber aestivum* was expected, since truffles are known to form ectomycorrhizae with a wide range of mycorrhizal plant partners such as *Betula, Carpinus, Castanea, Cedrus, Corylus, Fagus, Ostrya, Picea, Pinus, Pecan, Quercus, Tilia* ^2,3,13,46,68^. The mycorrhization level of silver fir was comparable to angiosperm hosts used commercial mycorrhization ^19^. Based on our results, satisfactory mycorrhization rates, i.e. at least 25% all fine roots mycorrhized with the desired truffle species ^48^, were only achieved after 12 months of mycorrhization. This suggests that commercial mycorrhization should be adapted to this time frame, or better mycorrhization conditions ought to be sought. Quality mycorrhized seedlings are not sufficient for cultivation of truffles with desired plant partners unless climate, soil, and cultivation conditions for both partners in symbiosis are met at the level to favor their growth and fruiting ^16^. Fortunately, silver fir ^36^ and *Tuber aestivum* ^18,32^ are tolerant to a wide range of soil conditions, nutrient content and availability as well as pH levels, making their cultivation plausible. A confirmation that suitable conditions can be met in nature is the example of beech-silver fir dominated forests covering the Dinaric Alps, where *Tuber aestivum* was collected in suitable micro locations ^69–73^. High altitude limestone mountain chains are also potential areas for future *Tuber aestivum* cultivation with silver fir or in combination with other host partners.

### Effect of earthworms on mycorrhization of silver fir with the commercial truffle species *Tuber aestivum*

The effects of earthworms on mycorrhization of silver fir with *Tuber aestivum* were ambiguous. The early effect after 6 months was positive, either due to perturbation or improved spore germination. The effect turned insignificantly negative after 12 months. Inconsistency in effects were previously reported by several authors who recorded either positive, neutral or negative effects on mycorrhizal root colonization ^29,30,74^. Due to the low number of studies, we can only speculate on possible mechanisms behind the effects. The early positive effect on mycorrhization could be attributed to stimulation of fungal growth due to enhanced nutrient and organic matter mineralization and substrate perturbation caused by earthworms ^28,30^. Mixing of substrate where spores patchy rather than evenly distributed, could favor mycorrhization. The late (e.g. 12 months after inoculation) negative effect on mycorrhization was predominantly due to grazing on already mycorrhized fine roots. Grazing of mycorrhized fine roots, but not non-mycorrhized roots, is due to a combination of reasons, for example, nutrient limitation in mycorrhization substrate ^28^, low appreciation of resin-rich silver fir bare roots, and the fact that mycelium of fungi is nutrient rich and as such a tempting substrate for hungry earthworms ^65^.

Based on our results we cannot give a clear management suggestion for application of earthworms in truffle mycorrhization procedures. The early effect on mycorrhization and general effects on substrate mixing and nutrients availability is positive, while longer mycorrhization times under the highly nutrient-limited environment of mycorrhization pots may cause it to become negative, as was already observed by ^75–78^ Bringing together experiences from negative and positive effects to modify the mycorrhization process may be a solution that is worth further investigation.

## Conclusions

In this study we revealed a significant effect of mycorrhization on root biomass and morphogenesis, as significantly higher seedling root tip density and specific root tip density compared to control seedlings was observed after 1 year from spore inoculation, findings which confirm Hypothesis 1. Hypothesis 2 can also be confirmed, as the observed results revealed that silver fir is an appropriate plant host for commercial cultivation of *Tuber aestivum* truffles, as inoculated silver fir seedlings passed the criteria or parameters which are defined for certification of seedlings mycorrhized with *Tuber* species. Lastly, we hypothesized that earthworms would have a positive impact on mycorrhizal root colonization as was reported by some authors; however, in our study earthworms had a positive short-term effect while the effect turned negative due to the secondary effect of grazing on ectomycorrhizal root tips, thus Hypothesis 3 remains unresolved.

## Supplementary Information

Supplementary Table 1.

## Data Availability

Reference collections for truffle samples used in the mycorrhization process are deposited at the LJF official herbarium. Original observations dataset generated and analyzed during the current study are available from the corresponding author on reasonable request.
