# Supplementary material for: Effect of earthworms on mycorrhization, root morphology and biomass of silver fir seedlings inoculated with black summer truffle (Tuber aestivum Vittad.)
Source: Sci Rep. 2021 Mar 17;11:6167. doi: 10.1038/s41598-021-85497-8 (PMC7971050; doi:10.1038/s41598-021-85497-8)
Supplement: Supplementary file 1 — Supplementary Table 1. [file 41598_2021_85497_MOESM1_ESM.docx]

**Effect of earthworms on mycorrhization, root morphology and biomass of silver fir seedlings inoculated with black summer truffle (*Tuber aestivum* Vittad.)**

Tina Unuk Nahberger^1^, Gian Maria Niccolò Benucci^2^, Hojka Kraigher^1^, Tine Grebenc^1^*

^1^ Slovenian Forestry Institute, Večna pot 2, 1000 Ljubljana

^2^ Department of Plant, Soil, & Microbial Sciences, Michigan State University, 426 Auditorium Road, East Lansing, MI 48824, USA

Supplement Table 1. p-values after a two-way ANOVA test for testing the effects of mycorrhization with *Tuber aestivum* (M), *Eisenia fetida* earthworms (E) and the combination of both treatments (M+E) against controls, on silver fir root biomass and fine root morphology parameters after 6 and 12 months of mycorrhization in containers under controlled conditions. ** - significance at 0.01, *** - significance at 0.001.

| Time after inoculation | Treatment | Fine root biomass  (mg) | Specific fine root length  (cm/g) | Fine root tip density  (no. of root tips/cm) | Specific fine root tip density  (no. of root tips/mg) | Branching density  (no. of branches/mg) |
| --- | --- | --- | --- | --- | --- | --- |
| 6 months | M | **0.010**** | 0.536 | 0.184 | 0.751 | 0.109 |
|  | E | 0.126 | 0.737 | 0.511 | 0.221 | 0.999 |
|  | M+E | 0.909 | 0.646 | **0.001***** | 0.071 | 0.555 |
| 12 months | M | 0.323 | 0.124 | **0.011**** | **0.001***** | 0.407 |
|  | E | 0.767 | 0.756 | 0.230 | 0.186 | 0.555 |
|  | M+E | 0.173 | 0.751 | 0.860 | 0.673 | 1.00 |
